# Elevation of sE-Selectin Levels 2–24 Months following Gestational Diabetes Is Associated with Early Cardiometabolic Risk in Nondiabetic Women

**DOI:** 10.1155/2012/278050

**Published:** 2012-04-22

**Authors:** Alina Sokup, Barbara Ruszkowska, Barbara Góralczyk, Krzysztof Góralczyk, Marek Szymański, Marek Grabiec, Danuta Rość

**Affiliations:** ^1^Department of Gastroenterology, Angiology and Internal Diseases, Nicolaus Copernicus University, Dr J. Biziel University Hospital, Bydgoszcz, Poland; ^2^Department of Endocrinology, Dr J. Biziel University Hospital, Bydgoszcz, Poland; ^3^Department of Pathophysiology, Nicolaus Copernicus University, Dr A. Jurasz University Hospital, Bydgoszcz, Poland; ^4^Department of Obstetrics and Gynecology, Nicolaus Copernicus University, Dr J. Biziel University Hospital, Bydgoszcz, Poland

## Abstract

*Objective*. We hypothesised that the endothelial dysfunction is associated with early glucose dysregulation and/or atherosclerosis risk factors in nondiabetic women with a previous history of gestational diabetes (pGDM). *Material/Methods*. Anthropometric parameters, glucose regulation (OGTT), insulin resistance (HOMA), lipids, biomarkers of endothelial dysfunction, and inflammation were evaluated in 85 women with pGDM and in 40 controls 2–24 months postpartum. *Results*. The pGDM group consisted of 67% normoglycemic women (pGDM-N) and 33% with prediabetic state (pGDM-P). The BMI, waist circumference, fasting and 2 h glucose (OGTT), soluble adhesion molecules, tissue plasminogen activator antigen, high sensitivity C-reactive protein, total-, LDL-cholesterol, and triglycerides/HDL-cholesterol ratio were higher in the pGDM women compared with the controls. After adjustment for BMI and fasting glucose, only higher triglycerides, higher TG/HDL and lower HDL-cholesterol were associated with pGDM. The pGDM-P differed from pGDM-N for only higher triglycerides and TG/HDL. The plasma level of sE-selectin was not independently associated with glucose concentration in pGDM group. sE-selectin level correlated with triglycerides, TG/HDL, plasminogen activator inhibitor-1 antigen, and sICAM-1. *Conclusions*. sE-selectin level correlated with components of metabolic syndrome, but only the atherogenic lipid profile was independently associated with a previous history of GDM in nondiabetic women 2–24 months postpartum.

## 1. Introduction

Women with the previous history of gestational diabetes mellitus (pGDM) have a significantly increased risk of type 2 diabetes and cardiovascular disease in the next years after delivery [1]. Very important problem is both GDM and its future consequences are drastically increasing public health and global problem which requires specific preventive strategies. 

In the years postpartum, women with pGDM have an increased cardiometabolic derangement, including vascular risk factors and early vascular dysfunction [2–4]. Several studies performed early (6 weeks-2 years) after delivery have found the association of cardiovascular risk factors with glucose intolerance, and in particular with type 2 diabetes [5, 6].

Shah et al. [1] have observed that there is an increased prevalence of metabolic syndrome in the third month postpartum among women with even mild glucose intolerance during pregnancy. Thus, it is possible that different stages of glucose dysregulation diagnosed during pregnancy are associated with early cardiovascular risk postpartum [7]. It has been suggested that the association between prediabetic state or diabetes and cardiovascular disease may be mediated through endothelial dysfunction [8, 9]. 

Soluble forms of adhesion molecules are released from shedding or proteolytic cleavage from the endothelial cell surface and may reflect overexpression of their respective membrane-bound forms [10]. Circulating levels of some endothelial-derived factors, soluble cell adhesion molecules, von Willebrand factor (vWF), tissue plasminogen activator (t-PA), and plasminogen activator inhibitor type1 (PAI-1) have been linked to the risk of type 2 diabetes in populations without pGDM [8, 11–13]. There are few controversial data concerning the associations between levels of soluble adhesion molecules and glucose regulation status in women with the pGDM. Studies simultaneously have assessed the associations with soluble adhesion molecules and hemostatic endothelial activation biomarkers with prediabetic and proatherogenic state in women with the pGDM [14–16].

In current study we hypothesized that early (2–24 months) endothelial dysfunction is associated with the development of early glucose dysregulation and/or the other atherosclerosis risk factors and may be the potential target included in preventive strategies in women with the pGDM. The aim of current study was to assess selected parameters of dysfunction of endothelium such as concentration of soluble E-selectin (sE-selectin), soluble vascular cell adhesion molecule-1 (sVCAM-1), and soluble intracellular adhesion molecule-1 (sICAM) in women with pGDM 2–24 months postpartum.

## 2. Material and Methods

### 2.1. Patients and Subjects

The research was conducted at the Diabetes and Pregnancy Unit at the Dr. J. Biziel University Hospital in Bydgoszcz, Poland, between 2005 and 2007.

A total of 85 Caucasian women with single gestations complicated by previous GDM (pGDM) and 40 women with normal glucose status during pregnancy (previous normal glucose tolerance—pNGT) as the control group were evaluated in the period of 2–24 months after delivery.

All subjects ingested at least 150 g carbohydrates a day and refrained from exercise for at least 3 days before the study. None of the participants had renal, hepatic, or cardiovascular disease. None of them were taking medications that affected lipid or carbohydrate metabolism. Women with a family history of diabetes mellitus, in addition of infectious processes, under stress as well as, those who were smoking, were not included.

GDM was diagnosed according to the modified criteria of the World Health Organization [17] after performing a fasting and 2 h 75 g oral glucose tolerance test (OGTT). These modified criteria are commonly used in Poland and are based on taking fasting glucose concentration of <5.6 mmol/L as a norm. This test was carried out in all pregnant women with a screening test (GCT-50-g oral glucose challenge) showing 2 h glucose value equal to or above 7.8 mmol/L. The screening test was performed during the first prenatal visit in all study groups and was repeated in the 24–28 weeks of gestation.

At the postpartum assessment, all participants underwent a physical examination that included anthropometric measurements. Waist circumference was measured at the smallest circumference between the ribs and iliac crest. BMI was calculated as weight in kilograms divided by the squared height in meters.

The standard 75 g 2 h OGTT was performed in order to assess glucose tolerance after delivery. The current status of glucose regulation was defined according to the modified WHO recommendations.

Venous blood (4.5 mL) for endothelial markers tests was collected in a fasting state into cooled tubes (Becton Dickinson Vacutainer System, Plymouth, UK) containing 0.13 mol/L trisodium citrate (the final blood-anticoagulant ratio was 9 : 1) after 30 min of rest between 7.00 and 9.00 am and after a 12 h overnight fast. The blood samples were immediately mixed and centrifuged at 3000 × g at +4°C for 20 min. The obtained platelet-poor plasma was divided into 200 *μ*L Eppendorf-type tubes, and then samples were frozen at −86°C until assayed, but no longer than six months. Blood for serum lipids; insulin concentration, and high sensitivity C-reactive protein (hsCRP) was collected in a tube containing no anticoagulant (Becton Dickinson Vacutainer 17490, Plymouth, UK), and the serum was separated by centrifuging at 2500 × g for 15 min and kept at +4°C until analyzed.

Serum glucose levels in samples from the OGTT were analyzed using the glucose hexokinase method on the automatic analyzer (Olympus Diagnostica GmbH, Ireland). Serum insulin concentration was determined using an immunochemical assay (8K41 Architect Insulin, Abbott Diagnostics, Denka Seiken, Japan) performed in the Architect Insulin analyzer (Abbott Diagnostics, Denka Seiken, Japan). Serum concentrations of triglycerides and total LDL and HDL cholesterol were measured by enzymatic techniques (Olympus Diagnostica GmbH, Ireland) on the Olympus automatic analyzer (Olympus Diagnostica GmbH, Ireland).

The concentration of t-PA:Ag was determined by Enzyme-Linked Immunosorbent Assay (ELISA)- ASSERACHROM t-PA (Diagnostica Stago, Asnieres, France), and PAI-1:Ag was determined by ELISA-ASSERACHROM PAI-1-(Diagnostica Stago, Asnieres, France), vWF:Ag was assessed by ELISA-STA-Liatest VWF:Ag (Diagnostica Stago, Asnieres, France), C-reactive protein (CRP) was measured with a high-sensitivity assay by test IMUCLONE CRP (hs) ELISA, C-Reactive Protein-ADI, using reagents from the American Diagnostica. Soluble sICAM-1 and sVCAM-1 were measured by using reagents from Bender Medsystems (Biomedica, Poland), and soluble E-selectin was determined by testing E-selectin (ELAM-1) antibody using reagent from IBL (Hamburg, Germany).

The homeostasis model assessment of insulin resistance (HOMA-IR) index was calculated as a sensitivity index of the insulin sensitivity of the whole body. The HOMA index was calculated by multiplying the fasting glucose level (millimoles per liter) by the fasting insulin level (microunits per milliliter) and dividing the product by 22.5 [18].

### 2.2. Statistical Methods

The statistical analysis was carried out using Statistica 8,0 (StatStoft, Cracow, Poland). The analyzed parameters were tested for normal distribution using the Shapiro-Wilk test. Variables were not normally distributed; *U* Mann Whitney test was used. Results shown are median (interquartile range) or frequencies (percentages). These results were further adjusted for BMI and fasting glucose. The *P* values <0.05 were considered statistically significant. Spearmans correlation coefficients were calculated to determine if there were associations between measured parametric values and sE-selectin level in the group with the history of GDM. To investigate the association between glucose level and plasma levels of biomarkers of endothelial dysfunction, all pGDM women were divided into two subgroups in accordance with low and high-glucose concentration within the prediabetic range. The high-glucose subgroup comprised of women with fasting glucose equal or above 6.4 mmol/L and <7.8 mmol/L at 2 h after 75 g glucose challenge (OGTT), women with fasting glucose <5.6 mmol/L and >8.9 mmol/L at 2 h, and women with fasting glucose equal to or above 6.4 mmol/L and >8.9 mmol/L at 2 h. Other pGDM women were included into the low-glucose subgroup. We compared plasma levels of endothelial dysfunction biomarkers in these subgroups. Additionally, the association of sE-selectin concentration with fasting glucose was examined by multiple logistic regression analysis.

### 2.3. Results

At the postpartum assessment of 85 women with pGDM and 40 women with normal glucose regulation during pregnancy (previous normal glucose tolerance—pNGT group), 7 women (8.24%) in pGDM group had impaired fasting glucose with normal glucose tolerance at 2 h OGTT (IFG/NGT), 16 (18.82%) normal fasting glucose with impaired glucose tolerance at 2 h OGTT (NFG/IGT), 5 (5.88%) impaired fasting glucose with impaired glucose tolerance at 2 h OGTT (IFG/IGT), and 57 women (67.06%) with normal glucose regulation (NFG/NGT). In the pNGT group all women were classified as NFG/NGT.

Women in pGDM group had significantly higher levels of fasting glucose, 2 h (OGTT) glucose, HbA1c, sVICAM-1, sICAM-1, sE-selectin, tPA:Ag, BMI, waist circumference, total cholesterol, LDL-cholesterol, triglycerides, and triglycerides/HDL-cholesterol ratio and lower HDL-cholesterol. After adjustment for BMI and fasting glucose, the pGDM was associated only with higher sVCAM-1, higher triglycerides, lower HDL-cholesterol, and higher triglycerides/HDL-cholesterol ratio (Tables 1 and 2).

Women in pGDM-P subgroup differed from pGDM-N group for higher fasting, 2 h (OGTT) glucose and higher triglycerides and triglycerides/HDL-cholesterol ratio (Table 2).

Only sE-selectin levels were significantly higher in high-glucose subgroup compared with the low-glucose subgroup, but this result was eliminated after further adjustment for BMI (Table 3). In multivariate regression analysis fasting glucose was associated only with insulin (*P* = 0.0087 and HOMA-IR (*P* = 0.0013).

In pGDM group sE-selectin was significantly correlated with triglycerides (*r* = 0.32), triglycerides/cholesterol-HDL ratio (*r* = 0.2910), PAI-1:Ag (*r* = 0.2647), tPA:Ag (*r* = 0.2622), and sICAM-1 (*r* = 0.2504).

## 3. Discussion

Among the 85 apparently healthy women with the previous history of gestational diabetes (pGDM), 33% showed prediabetic state; 19% impaired glucose tolerance, 8% impaired fasting glucose and 6% impaired fasting glucose with impaired glucose tolerance at two hours of oral glucose tolerance test. These results are in accordance with previous reports [5].

Our study shows that these nondiabetic women with pGDM have higher concentrations of cardiovascular risk factors at average 10 months assessment after delivery in comparison with women with normal glucose regulation during pregnancy. The pGDM population was characterized by higher values of body fat indexes as well as levels of endothelial dysfunction parameters (sICAM-1, sVCAM-1, sE-selectin, tPA:Ag) and low-grade inflammation (hsCRP) which coexisted with dyslipidemic lipids profile and comparable insulin resistance. After adjustment for BMI and fasting glucose, only higher triglycerides, higher sVCAM-1, lower HDL-cholesterol, and higher triglycerides/HDL-cholesterol ratio were associated with pGDM.

Thus, our results suggest that the atherogenic dyslipidemia my be specific and important metabolic feature of nondiabetic women with pGDM during first years pospartum. It is well known and generally accepted that this metabolic abnormality is associated with endothelial dysfunction and insulin resistance [19–21]. Interestingly, our current study shows that atherogenic lipids profile in pGDM nondiabetic women is independent of both insulin resistance and BMI.

Our results are inconsistent with one previous report suggesting elevated sE-selectin levels during 12–26 months after delivery only in pGDM women with abnormal glucose regulation and with other two studies presenting higher sE-selectin levels irrespectively of glucose regulation status [14–16] and metabolic abnormalities related to insulin resistance [16]. Moreover, results of these studies suggest that elevated level of sE-selectin in post-GDM women is independent of time since delivery [14, 16].

The current study is the first in which we observed higher plasma levels of sICAM-1, sE-selectin, and tPA:Ag levels with simultaneously normal levels of PAI-1:Ag and vWF:Ag in nondiabetic pGDM women with both abnormal and normal glucose regulation.

Several studies have shown that circulating levels of ICAM-1, E-selectin [8, 11], tPA:Ag [12, 13], vWF:Ag, and PAI-1:Ag [12] have been linked to the risk of type 2 diabetes in populations without the previous history of GDM.

In our study we have found that in the case of nondiabetic women with pGDM, high-glucose concentrations within the prediabetic range were associated with higher sE-selectin levels. This association was eliminated after further adjustment for BMI. Additionally, in multivariate regression analysis, fasting glucose was associated only with insulin concentration and insulin resistance assessed by HOMA-index. Thus, our results showed that endothelial dysfunction evaluated by soluble forms of its specific biomarkers was not independently associated with glucose dysregulation in nondiabetic women with pGDM.

 In the whole population of pGDM women, sE-selectin levels correlated positively with other markers of endothelial dysfunction, tPA:Ag, sICAM-1, and with surrogate markers of insulin resistance, triglycerides, triglycerides/HDL-cholesterol ratio, and PAI-1 : Ag level. Recently, triglycerides/HDL cholesterol ratio has been suggested to be a predictor of insulin resistance and of the proportion of small and dense LDL particles, which is characteristic of atherogenic phenotype of the metabolic syndrome [22].

Results of our study suggest also that endothelial dysfunction at assessment 2–24 months (average 10 months) after delivery in nondiabetic women with pGDM is characterized by higher plasma levels of circulating ICAM-1, sE-selectin, and tPA:Ag in women with normal glucose regulation as well as pGDM women with prediabetic state, matched for age, body fat indexes values, inflammation, and PAI-1:Ag level. These observations agree with the previous studies of Bo et al. performed 6.5 years after delivery in pGDM women. In this study, they obtained higher levels of sE-selectin and sICAM-1 and higher intima media thickness (IMT) values characterized pGDM women, even those without any components of the metabolic syndrome except for glycaemia [16]. In all pGDM women, sE-selectin, sICAM-1, interleukin-6, and hsCRP values were significantly associated with IMT, thus supporting associations of these biomarkers with pathogenesis of atherosclerosis [16].

Our present findings contrast with the results of previous study of Lawrence et al. [15]. These authors have found clinical and biochemical markers of insulin resistance but have not shown any associations between sE-selectin levels and these markers [15]. Additionally, higher levels of sE-selectin in post-GDM women were restricted to women with abnormal glucose regulation. Thus, discrepant results of current study and the study of Lawrence et al. may suggest pathophysiological differences in populations subject to examinations [15].

## 4. Conclusion

Higher body fat indexes, glucose during OGTT, levels of endothelial dysfunction biomarkers, low-grade inflammation (hscRP), and atherogenic dyslipidemia characterized nondiabetic women with pGDM and normal glucose regulation as well as women with pGDM and prediabetic state assessed 2–24 months after delivery. Our findings indicated that only parameters of atherogenic dyslipidemia and sVCAM-1 level were associated with pGDM independently of BMI, insulin resistance, and fasting glucose. sE-selectin levels correlated with metabolic syndrome components but were not independently associated with abnormal glucose regulation. Thus, the atherogenic dyslipidemia may be the key metabolic feature and a predictor of cardiovascular risk in women with pGDM and thus needs early and regular monitoring and special therapeutic interventions after an index pregnancy.

## Figures and Tables

**Table 1:** Characteristics of women with previous history of GDM (pGDM group) and control participants with normal glucose regulation during pregnancy (pNGT group).

Variables (units)	pGDM (*n* = 85)	pNGT (*n* = 40)	*P-*values
Age (years)	29.00 (26.00, 35.00)	27.00 (25.00, 35.50)	0.2040
BMI (kg/m^2^)	23.68 (20.96, 27.54)	22.00 (20.31, 24.33)	0.0098
Waist (m)	0.80 (0.73–0.92)	0.74 (0.69–0.78)	0.0007
HbA_1_C (%)	5.50 (5.40–5.70)	5.40 (5.30–5.40)	0.4399*
Fasting plasma glucose (mmol/L)	4.50 (4.61–5.33)	4.72 (4.50–4.78)	0.0001*
2-h (OGTT) plasma glucose (mmol/L)	6.16 (5.55–7.66)	4.84 (4.77–5.44)	0.1655*
Insulin (pmol/L)	54.87 (41.67–80,56)	63.20 (54.87–71.53)	0.1755
sVCAM-1 (ng/mL)	1113.53 (419.60–1616.70)	672.05 (455.70–1113.46)	0.00018*
sICAM-1 (ng/mL)	294.89 (238.73–364.19)	140.40 (121.34–179.90)	0.5053*
sE-selectin (ng/mL)	28.13 (19.17–43.95)	20.90 (17.11–26.01)	0.0791* 0.4791**
tPA:Ag (ng/mL)	4.89 (3.53–7.66)	3.50 (2.66–4.64)	0.4058*
vWF:Ag (%)	104.30 (96.20–122.30)	104.87 (74.96–125.67)	0.4405
PAI-1:Ag (ng/mL)	57.09 (46.28–79.43)	71.36 (50.54–81.80)	0.1371
hsCRP (*μ*g/mL)	1.22 (0.66–2.76)	0.41 (0.22–0.79)	<0.0004* 0.1939**
Total-cholesterol (mmol/L)	5.04 (4.55–5.51)	4.56 (4.27–5.09)	0.9940*
HDL-cholesterol (mmol/L)	1.53 (1.28–1.73)	1.73 (1.56–1.82)	0.0027* 0.0449**
LDL-cholesterol (mmol/L)	3.10 (2.79–3.54)	2.57 (2.30–3.05)	0.4022*
Triglyceride (mmol/L)	0.97 (0.78–12.83)	0.86 (0.67–1.05)	0.0006* <0.0001**
Triglyceride/HDL cholesterol	1.52 (1.06–2.63)	1.11 (0.89–1.50)	<0.0001* <0.0001**
HOMA-IR (%)	1.67 (1.22–2.80)	1.89 (1.58–2.37)	0.3973
NFG/IGT *n* (%)	16 (18.82)	—	—
IFG/NGT *n* (%)	7 (8.24)	—	—
IFG/IGT *n* (%)	5 (5.88)	—	—
NFG/NGT *n* (%)	57 (67.06)	40 (100)	—

Values are median (IQR) or *n* (%). *P* values were from *U*-Mann Whitney test. *adjusted for body mass index, **adjusted for fasting glucose.

**Table 2:** Clinical and laboratory data for participants in studied groups: pGDM-P, pGDM-N and pNGT as a control group.

Variable (units)	pGDM-P *n* = 29	pGDM-N *n* = 56	pNGT *n* = 40	*P-*values
Age (years)	31.50 (27.50–35.50)	30.00 (26.00–35.00)	27.00 (25.00–35.50)	0.1145
BMI (kg/m^2^)	25.12 (21.83–29.04)^b^	24.44 (21.26–27.22)^a^	22.00 (20.31–24.33)	0.0022
Waist circumference (cm)	80.00 (73.00–96.00)^b^	82.00 (73.00–90.00)^a^	74.50 (69.00−78.00)	0.0010
Fasting plasma glucose (mmol/L)	5.16 (4.77−5.88)^b,c^	4.77 (4.44−5.13)	4.72 (4.50−4.78)	0.0002
2-h (OGTT) plasma glucose (mmol/L)	8.27 (8.05−8.77)^b,c^	5.88 (5.44–6.66)^a^	4.84 (4.77–5.44)	<0.0001
Insulin (pmol/L)	57.44 (42.36–95.84)^cc^	54.17 (41.67–69.45)	63.20 (54.87–71.53)	0.0353
sVCAM-1 (ng/mL)	960.30 (353.73–1638.03)	1127.63 (466.68–1582.58)^a^	672.05 (455.70–1113.46)	0.0553
sICAM-1 (ng/mL)	295.90 (238.80–363.38)^b^	299.28 (243.46–364.19)^a^	140.40 (121.34–179.90)	<0.0001
sE-selectin (ng/mL)	35.80 (26.37–43.65)^b^	28.57 (20.35–42.54)^a^	20.90 (17.11–26.01)	0.0022
tPA:Ag (ng/mL)	6.01 (4.09–7.51)^b^	4.68 (3.25–7.24)^a^	3.50 (2.66–4.64)	0.0005
vWF:Ag (%)	107.79 (96.20–131.59)	104.30 (95.36–117.22)	104.87 (74.96–125.67)	0.6498
PAI-1:Ag (ng/mL)	57.67 (49.44–83.75)	57.09 (42.85–85.12)	71.36 (50.54–81.80)	0.2178
hsCRP (*μ*g/mL)	1.30 (0.81–2.86)^b^	1.32 (0.66–2.76)^a^	0.41 (0.22–0.79)	<0.0001
Total-cholesterol (mmol/L)	5.33 (4.84–5.92)^b^	5.02 (4.50–5.48)^a^	4.56 (4.27–5.09)	0.0022
HDL-cholesterol (mmol/L)	1.45 (1.29–1.73)^b^	1.55 (1.27–1.73)^a^	1.73 (1.56–1.82)	0.0048
LDL- cholesterol (mmol/L)	3.26 (2.97–4.06)^b^	3.03 (2.77–3.54)^a^	2.57 (2.30–3.05)	<0.0001
Triglyceride (mmol/L)	1.21 (1.02–2.22)^b,c^	79.00 (63.00–116.00)	74.50 (58.50–91.00)	0.0004
Triglyceride/HDL cholesterol	1.83 (1.35–4.06)^b,c^	1.33 (0.95–2.30)	1.11 (0.89–1.50)	0.0003
HOMA-IR (mmol/L)	2.32 (1.27–3.49)^ccc^	1.64 (1.22–2.15)	1.89 (1.58–2.37)	0.0499

*P*-values for testing over the three groups (ANOVA): a: *P* < 0.05 controls versus previous GDM with normal glucose regulation; b: *P* < 0.05 controls versus previous GDM with prediabetic state; c: *P* < 0.05 previous GDM with normal glucose status versus previous GDM with prediabetic state, cc: *P* = 0.05, ccc: *P* = 0.06.

**Table 3:** Plasma concentrations of endothelium markers according to low and high-glucose concentration within the prediabetic range.

Variable (units)	Low glucose (*n* = 58)	High glucose (*n* = 27)	*P-*values
sVCAM (ng/mL)	849.78 (550.70–1285.55)	669.18 (419.60–13.90)	0.3610
sICAM (ng/mL)	222.44 (152.66–317.08)	247.79 (212.59–338.68)	0.1066
sE-selectin (ng/mL)	25.90 (19.03–40.17)	37.75 (27.93–46.15)	0.0314 0.0791*
vWF:Ag (%)	101.72 (91.76–119.04)	107.10 (86.68–136.34)	0.3457
tPA:Ag (ng/mL)	4.05 (2.63–6.05)	4.92 (3.69–6.68)	0.0838
PAI-1:Ag (ng/mL)	61.85 (47.42–85.18)	55.47 (44.93–83.75)	0.7013

Values are median (IQR), and *P* values were from *U* Mann Whitney test. *Adjusted for body mass index.

## Abbreviations

pGDM: Previous gestational diabetes
sVCAM-1: Soluble vascular cell adhesion molecule-1
sICAM: Soluble intracellular adhesion molecule-1
PAI-1:Ag: Plasminogen activator inhibitor-1 antigen
tPA:Ag: Tissue plasminogen activator antigen
hsCRP: High sensitivity C-reactive protein.
